# *Relative Reduction in Prevalence* (*RRP*): An Alternative to Cohen’s Effect Size Statistics for Judging Alcohol, Cigarette, and Marijuana Use Prevention Outcomes

**DOI:** 10.1007/s10935-020-00608-x

**Published:** 2020-08-28

**Authors:** William B. Hansen

**Affiliations:** Prevention Strategies, Greensboro, NC USA

**Keywords:** Effect size, Alcohol, Cigarette, Marijuana, Prevention, Evaluation

## Abstract

Jacob Cohen developed two statistical measures for judging the magnitude of effects produced by an intervention, known as Cohen’s *d*, appropriate for assessing scaled data, and Cohen’s *h*, appropriate for assessing proportions. These have been widely employed in evaluating the effectiveness of alcohol, cigarette, marijuana, and other drug prevention efforts. I present two tests to consider the adequacy of using these statistics when applied to drug use prevention programs. I used student survey data from grades 6 through 12 (*N* = 1,963,964) collected by the Georgia Department of Education between 2015 and 2017 and aggregated at the school level (*N* = 1036). I calculated effect sizes for an imaginary drug prevention program that (1) reduced 30-day alcohol, cigarette, and marijuana prevalence by 50%; and (2) maintained 30-day prevalence at a pretest level for multiple years. While both approaches to estimating intervention effects represent ideal outcomes for prevention that surpass what is normally observed, Cohen’s statistics failed to reflect the effectiveness of these approaches. I recommend including an alternative method for calculating effect size for judging program outcomes. This alternative method, *Relative Reduction in Prevalence* (*RRP*), calculates ratio differences between treatment and control group drug use prevalence at posttest and follow-up, adjusting for differences observed at pretest. *RRP* allows researchers to state the degree to which an intervention could be viewed as efficacious or effective that can be readily understood by practitioners.

## Introduction

Preventing or deterring the onset of drinking alcohol, smoking cigarettes, and using marijuana and other drugs among adolescents has long been a priority throughout the developed world. The challenge facing researchers and program developers is creating interventions that demonstrate efficacy in critical tests and effectiveness once they are disseminated. The challenge for administrators and others is understanding the potential adopted programs have for reducing substance use.

Judging efficacy and effectiveness require the use of statistics for estimating the effect size magnitude. Researchers have historically relied on Cohen’s *d* or *h* (Cohen, 1988) to estimate the magnitude of effect. Cohen’s *h* is appropriate when data are proportional. For example, when prevention studies collect dichotomous (yes/no) responses and summarize across respondents, the proportion of cases who report use can be used to calculate *h*. Cohen’s *d* is appropriate for calculating effect size when scaled values are available, for instance when data being evaluated include such measures as average frequency or quantity of use. Because meta-analyses often transform impact estimates (e.g., *t* tests) provided in research publications into a common metric—the effect size (Glass, Smith, & McGaw, 1981; Ialongo, 2016)—it is not unusual for Cohen’s *d* to be used even when Cohen’s *h* would be the appropriate statistic.

Researchers have published numerous analyses that examine published randomized control trials and quasi-experimental studies of drug prevention. Literature reviews are typically distinguished by their lack of effect size statistics (Hansen, 1992; Skara & Sussman, 2003; Vickers, Thomas, Patten, & Mrazek, 2002). Meta-analyses, on the other hand, use effect size statistics to compare intervention efficacy across studies (Bangert-Drowns, 1988; Bruvold, 1990, 1993; Hwang, 2007; Hwang, Yeagley, & Petosa, 2004; Kok, van den Borne, & Mullen, 1997; Porath-Waller, Beasley, & Beirness, 2010; Rooney & Murray, 1996; Shamblen & Derzon, 2009; Tobler, 1986, 1997; Tobler et al., 2000; Tobler & Stratton, 1997; Wilson, Gottfredson, & Najaka, 2001). The final category of research summary, systematic reviews (Foxcroft, Ireland, Lister‐Sharp, Lowe, & Breen, 2003; Foxcroft, Lister‐Sharp, & Lowe, 1997; Foxcroft & Tsertsvadze, 2012), evaluates the efficacy of drug prevention interventions after screening out methodological weaknesses. Meta-analyses often screen for methodological quality, while systematic reviews often include quality measures, but do not always screen out weak studies. Several reviews, meta-analyses, and systematic reviews have also specifically targeted understanding program components that account for differences among outcomes (Cuijpers, 2002a, 2002b; Dobbins, DeCorby, Manske, & Goldblatt, 2008; Hansen, 1992).

Among the 19 meta-analyses and systematic reviews cited above, nine provided no documentation about the specific methods used for calculating effect size. All remaining reports reference Cohen’s *d*. All but one of these also reference additional methods. These include adjustments proposed by Hedges (1984) and Hedges and Olkin (2014) added effect size estimates based on the transformation of non-effect size statistical values (Glass, Smith, & McGaw, 1981; Ialongo, 2016). Only five meta-analyses (Tobler, 1986, 1997; Tobler et al., 2000; Tobler & Stratton, 1997; Wilson et al., 2001) specifically mention using Cohen’s *h* to estimate effect size.

Cohen proposed conventions for interpreting effect size. An effect size of 0.2 would be considered to reflect a “small” effect, one of 0.5 would be considered to reflect a “moderate” effect, and an effect size above 0.8 would be considered a “large” effect. In reference to this standard, Cohen noted, “Although arbitrary, the proposed conventions will be found to be reasonable by reasonable people” (1988, p. 13). In discussing this, Cohen avoids strictly applying this standard, noting that each field should develop interpretations appropriate to its topic of study. However, when interpretations of prevention efficacy are made, they frequently refer to Cohen’s conventions. For example, among the prevention meta-analyses cited above, several (Hwang et al., 2004; Kok et al., 1997; Porath-Waller et al., 2010; Rooney & Murray, 1996; Tobler et al., 2000) specifically reference these specific cut points in interpreting findings. Other meta-analyses (Fagan & Catalano, 2013; Foxcroft et al., 1997, 2003; Foxcroft & Tsertsvadze, 2012; Hwang, 2007), without specifically citing these conventions, appear to have fully adopted Cohen’s cut points based on the way they interpreted their results.

In this paper, I argue that Cohen’s effect size statistics are often inappropriate for evaluating changes in prevalence produced by adolescent drug prevention programs. Other researchers (Greenberg & Abenavoli, 2017) have made a similar argument. My argument focuses on a bias for minimizing effects when base rate prevalence is low, which is often the case in prevention research. I examine Cohen’s effect size estimates relevant to adolescent alcohol, tobacco, and marijuana use prevention. I use an existing large database of student surveys to calculate effect size from several perspectives using hypothetical ideal prevention outcomes to demonstrate the challenges of relying solely on Cohen’s effect size statistics and his published conventions. I offer an alternative effect size approach, *Relative Reduction in Prevalence* (*RRP*), to interpret prevention program outcomes. I contrast *RRP* to Cohen’s *h* and a statistic proposed by Skara and Sussman (2003), *Percentage Reduction (PR)*.

## Method

### Source of Data

The Georgia Department of Education routinely administers surveys to 6th through 12th grade students. I selected student survey data collected between 2015 and 2017 for analysis. The dataset consisted of 1,960,830 surveys collected from students enrolled in 1036 schools. Data include reports of past 30-day alcohol, cigarette, and marijuana use. Alcohol, cigarette, and marijuana use were dichotomized with “non-use” coded as a zero (0) and “use” coded as a one (1).

### Procedures

In this paper, I complete a thought experiment. This approach assumes that none of the artifacts that plague real-life research (Cheung & Slavin, 2016) need to be accounted for. This study relies on actual data from Georgia students but involves the creation of an imaginary intervention that has the ability to: (1) reduce substance use prevalence by 50%, and (2) eliminate any new future substance use onset in later grades. Observed data from Georgia serve as the control group, and the treatment group behaviors reflect these hypothetical outcomes.

### Formulae

#### Cohen’s *h*

I calculated behavior-specific effect size, using proportions of students reporting past 30-day use (P), using Cohen’s *h* where *ϕ* for each condition is calculated using the formula:

$$\phi = 2\, \times \,arcsin\sqrt P$$

Cohen’s *h* is calculated:

$$h = \phi_{Control} - \phi_{Treatment}$$

The control condition, *ϕ*_*Control*_, consists of the observed Georgia prevalence rates for each grade and the treatment condition, *ϕ*_*Treatment*_, are the hypothetical improvements noted above.

#### Skara–Sussman’s *Percentage Reduction* (*PR*)

Because of the longitudinal nature of prevention research, Skara and Sussman (2003) recommended applying a formula that compares the pretest–posttest change in the treatment group (Δ*Treatment*) to the change in the control group (Δ*Control*) where each consists, respectively, of the percent of users at the posttest (or any subsequent follow-up) minus the percent of users at the pretest. *Percentage Reduction (PR)* is calculated:

$$PR = \Delta Treatment - \Delta Control$$

#### *Relative Reduction in Prevalence* (*RRP*)

I propose an alternative effect size statistic, *Relative Reduction in Prevalence* (*RRP*), that uses the terms from the Skara–Sussman formula to create an effect size estimate. This statistic compares the pretest–posttest changes in the prevalence in the treatment (Δ*Treatment*) group with that of the control (Δ*Control*) group where each consists, respectively, of the percent of users at the posttest (or any subsequent follow-up) minus the percent of users at the pretest.

$$RRP = 1 - \frac{\Delta Treatment}{\Delta Control}$$

## Results

### Cohen’s Effect Size for Behavioral Outcomes

**Fig. 1:**
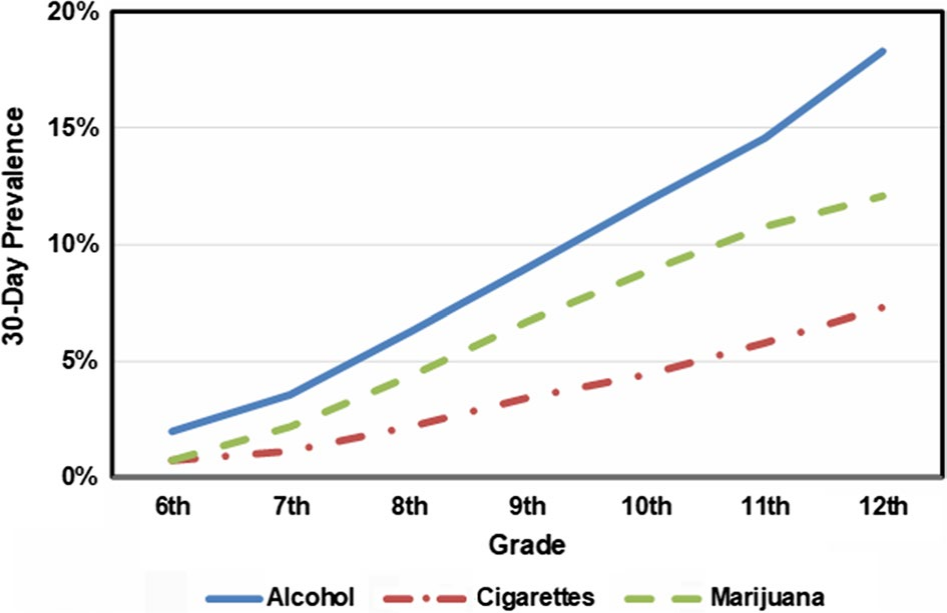
30-day prevalence of drinking alcohol, smoking cigarettes and using marijuana by grade averaged across schools in Georgia

Figure 1 presents results of past 30-day alcohol, cigarette, and marijuana use averaged across schools in Georgia. As would be expected from any such dataset, the past 30-day prevalence of drinking, smoking, and using marijuana increases grade-by-grade.

Table 1 presents effect size outcomes (Cohen’s *h*) when a hypothetical intervention could reduce the prevalence of alcohol, cigarette, and marijuana use by 50% at each grade. As typically implemented, these data assume that a hypothetical intervention would be delivered at an earlier grade and that effects would likely be observed in the subsequent year or years. Applying Cohen’s conventions, researchers would interpret the effects on alcohol to be “small” through at least 10th grade. The effects on cigarettes would be “small” throughout. Marijuana outcomes would be judged to be “small” until past the 11th grade.

**Table 1:** Observed effect size (Cohen’s *h*) should a hypothetical intervention reduce prevalence of use by 50%

Grade	Alcohol	Cigarettes	Marijuana
6th	0.08	0.04	0.04
7th	0.11	0.06	0.08
8th	0.15	0.08	0.12
9th	0.18	0.10	0.15
10th	0.21	0.12	0.18
11th	0.24	0.14	0.20
12th	0.27	0.16	0.21

An alternative way to think about assessing the effectiveness of prevention assumes that, as a result of a hypothetical intervention, no new cases emerge. In other words, such an intervention would completely suppress incidence at subsequent grades. This is farfetched because no intervention has achieved such outcomes long term. Table 2 presents the Cohen’s *h* effect size for the current dataset. For these results, as an example, an intervention delivered in 6th grade would maintain the same level of prevalence for alcohol (1.9%), cigarettes (0.5%), and marijuana (0.5%) throughout middle and high school years, whereas the prevalence rates for the hypothetical control group would increase as expected based on the increases observed in the Georgia data.

**Table 2:** Estimated effect size (Cohen’s *h*) for a hypothetical intervention that results in no subsequent increase in 30-day prevalence of use

Grade of implementation	Substance	Years of follow-up					
		1	2	3	4	5	6
	Alcohol						
6th		0.10	0.23	0.34	0.44	0.52	0.61
7th		0.12	0.23	0.34	0.42	0.51	
8th		0.11	0.21	0.30	0.39		
9th		0.10	0.19	0.28			
10th		0.09	0.18				
11th		0.09					
	Cigarettes						
6th		0.06	0.13	0.21	0.27	0.33	0.40
7th		0.07	0.15	0.21	0.27	0.34	
8th		0.08	0.14	0.20	0.27		
9th		0.06	0.12	0.19			
10th		0.06	0.13				
11th		0.07					
	Marijuana						
6th		0.13	0.26	0.37	0.46	0.52	0.57
7th		0.13	0.24	0.33	0.40	0.44	
8th		0.11	0.20	0.27	0.31		
9th		0.09	0.15	0.19			
10th		0.07	0.11				
11th		0.04					

After one year of implementation with no new use, the effect size for an alcohol prevention intervention at any grade would vary between 0.09 and 0.12. Cigarette interventions would fare worse with Cohen’s *h* effect size, varying between 0.06 and 0.08. Marijuana prevention intervention effect size would vary between 0.04 and 0.13. On average, an alcohol prevention program would need to succeed for about 2 years at restricting the onset of use to achieve a “small” effect size, with slightly better outcomes for interventions in 6th and 7th grades. Cigarette interventions would need to completely suppress incidence for three or more years. Marijuana prevention programs would need to completely suppress onset for 2 years if the intervention were initially pegged to the prevalence observed in 6th through 8th grades, and for three years if pegged to 9th grade prevalence. Effect size above 0.50 was observed only for interventions that completely suppressed alcohol use onset for five or more years and that maintained marijuana for 5 or 6 years at 6th grade rates.

### Comparisons Using *Relative Reduction in Prevalence* (*RRP*) and *Percentage Reduction* (*PR*)

A hypothetical set of outcomes is portrayed in Table 3 that demonstrates Cohen’s *h*, *RRP*, and *PR* values across multiple years of evaluation. Prevalence data in the table are not drug-specific and are generated by my imagination, but generally reflect trends I have observed in other studies. The posttest and follow-up periods are arbitrary but may be thought of as annual or semi-annual events. The changes over time in treatment condition prevalence are designed to reflect a strong intervention effect. These data also assume a small pretest difference between treatment and control conditions which is typical of many prevention studies.

**Table 3:** A demonstration of how effect size (Cohen *h*), *Relative* *Reduction* *in Prevalence* (*RRP*), and Skara–Sussman’s *Percentage Reduction* (*PR*) provide different outcomes for interpreting a hypothetical example of change in drug use behavior

	Prevalence		Cohen’s effect size			Alternatives			
	Control (%)	Treatment (%)	φControl	φTreatment	*h*	ΔControl (%)	ΔTreatment (%)	*RRP*	*PR* (%)
Pretest	1.86	2.00							
Posttest	3.47	2.32	0.37	0.31	0.07	1.61	0.32	0.80	− 1.29
Follow-up 1	6.11	4.09	0.50	0.41	0.09	4.25	2.09	0.51	− 2.16
Follow-up 2	9.01	6.04	0.61	0.50	0.11	7.15	4.04	0.44	− 3.11
Follow-up 3	12.11	8.11	0.71	0.58	0.13	10.25	6.11	0.40	− 4.14
Follow-up 4	15.10	10.12	0.80	0.65	0.15	13.24	8.12	0.39	− 5.12
Follow-up 5	18.46	12.37	0.89	0.72	0.17	16.60	10.37	0.38	− 6.23

Cohen’s *h* reflects outcomes similar to those presented in Tables 2 and 3; consistently “small” effect size as judged by Cohen’s conventions.^1^*RRP* reflects larger magnitudes of observed differences; effect size at posttest are “large” by Cohen’s conventions and decay gradually over time. This creates the pattern of Cohen’s *h* and Skara–Sussman’s *PR* increasing with successive follow-up surveys whereas *RRP* declines. Even so, the evidence of effectiveness based on the general size of the difference is more obvious when using the *RRP* statistic.

## Discussion

### Interpretation of Cohen’s Effect Size Findings

These analyses call into question the reasonableness of using Cohen’s effect size when applied to evaluating the impact of interventions on preventing the onset of drug use. In a practical sense, any alcohol, cigarette, or marijuana prevention program that could achieve a reduction of 50% in prevalence would be judged to be effective. However, Cohen’s effect size was very small for the first set of hypothetical intervention outcomes I modeled, particularly for middle school ages (6th, 7th, and 8th grades). While no data exist to prove the point, a reasonable person would likely conclude that an intervention that could consistently reduce substance use by even as much as 15–20% would be considered remarkably effective and worth the investment and time and materials. Yet Cohen’s effect size would be interpreted to show only “small” effects.

Similarly, any program that could result in the long-term complete suppression of onset would surely be judged to be effective. Yet, as modeled in the second set of analyses, it was only when the hypothetical intervention achieved the longest possible suppressed outcomes that effect size rose to the level of a “small” or “moderate” effect. Further, “small” and “moderate” effect sizes were then only observed for alcohol and marijuana. With an increasing base rate associated with age, an intervention that might suppress new cases for even one or two years would be considered to be effective by most practitioners. In practice, longitudinal outcomes may be significantly smaller than concurrent outcomes (Adachi & Willoughby, 2015), suggesting that it may be fundamentally challenging to achieve such long-term effects.

### An Alternative Measure of Effect Size

I tested an alternative statistical measure of effect size, *Relative Reduction in Prevalence (RRP)*. For drug prevention evaluations, *RRP* would be directly interpretable. It describes reductions in the onset of use attributable to the treatment in comparison to the control group. This would allow researchers to be able to state the degree to which an intervention could be viewed as efficacious or effective.

*RRP* is essentially a risk ratio with pretest values considered. It recognizes that it is the comparative pretest–posttest change in addition to the magnitude of difference between groups that is most relevant to understanding program efficacy or effectiveness.

One characteristic of *RRP* that makes it suitable for evaluating prevention programs is that it capitalizes on having longitudinal data. While there may be adjustments that researchers could adopt, Cohen’s *d* and *h* statistics do not account for pretest base rates or include change over time as a standard component. Typically, pretest values are simply assumed to be equivalent, which is rarely true in practice. Including pretest–posttest change scores as an essential component for estimating effect size is appropriate and adds value to understanding outcomes.

### Benchmarks

An essential element of Cohen’s effect size statistics that make outcomes interpretable is that Cohen also provided benchmark conventions. Because *RRP* is an alternative method for calculating effect size, Cohen’s conventions may be useful for interpreting observed results as well. However, some consideration should be given before a full-scale adoption of these conventions.

Prior research in education (Hill, Bloom, Black, & Lipsey, 2008; Lipsey et al. 2012) suggests that a variety of benchmarks other than Cohen’s conventions might be applied to interpret the substantive significance of outcomes. Included for consideration might be such factors as comparisons with known normative patterns of development and a comparison of prior effect size results. In both of these cases, there is a heavy reliance on prior research findings. Normative patterns of drug use onset are becoming increasingly available through national and statewide surveys. However, it is apparent that, despite the general year-after-year increases in prevalence, sub-populations differ markedly in their trajectories of onset, making the selection of reference data challenging. Similarly, based on outcomes from published meta-analyses and systematic reviews, effect size varies widely, and formal standards are difficult to establish.

One alternative criterion for interpreting outcomes involves establishing effect size cut points based on prior research and using clinical judgments by practitioners. Researchers examining issues with improving patient conditions in clinical settings have used “minimal clinically important differences” (MCID) as a means of assessing the potential of treatments to be worthy of consideration (Angst, Aeschlimann, & Angst, 2017; Copay, Subach, Glassman, Polly, & Schuler, 2007; Jaeschke, Singer, & Guyatt, 1989; King, 2011). For example, Cuijpers, Turner, Koole, Van Dijke, and Smit (2014) discussed the clinical relevance of Cohen’s conventions when considering interventions addressing depressive disorders. In analyses completed by this team, an effect size of 0.24 was deemed sufficient to interpret an intervention has being relevant and worthy of adoption. Having access to *RRP* estimates would make it easier for practitioners to gain an understanding of what would constitute an effective drug prevention program.

Several researchers have suggested that even a small effect size may be important (Caulkins, Pacula, Paddock, & Chiesa, 2004; Cuijpers, 2002a; Foxcroft & Tsertsvadze, 2012). This may be particularly true if programs with a smaller than ideal effect size can be widely disseminated and sustained over a long period of time. In cases where there is a small effect size, there may yet be important benefit–cost ratios attained to recommend program adoption (Miller, Hendrie, & Derzon, 2011). Interpretable effect size using *RRP* may assist in making such determinations.

My team is involved in developing a strategy that will compare treated students in a dissemination environment to algorithmically generated “virtual” controls for which comparisons of rates of prevalence would also be appropriate (Hansen, Chen, Saldana, & Ip, 2018). Presenting pretest–posttest prevalence rates and using the *RRP* to present percent differences between treatment and controls would provide information that could be readily interpretable by practitioners.

### Adjustments

Results presented in Table 3 reflect what might be thought of as the normal case where prevalence among treated cases increases more slowly than among controls. *RRP* works equally well when control group prevalence increases while treatment reduces prevalence. There are several cases, however, that require an adjustment.

1. If there is no change in control group prevalence, *RRP* cannot be calculated because a division by zero error occurs. In this case, Skara–Sussman *PR* and Cohen’s *h* are the only interpretable statistics.
2. If both treatment and control have reductions in prevalence, for example if pretest-to-posttest reductions in control and treatment were respectively − 0.07% and − 0.14%, *RRP* would be − 1.00. Reversing the divisor and dividend (switching Δ*Treatment* and Δ*Control*) results in an appropriate solution resulting in an *RRP* of 0.50.
3. A similar solution is needed if prevalence in the control group reduces and prevalence in the treatment group increases. For example, if pretest-to-posttest reductions in control and treatment were respectively − 0.07% and + 0.14%, *RRP* would be 3.00. Switching the divisor and dividend results in an *RRP* of − 1.50, which is an appropriate solution.
4. If the control prevalence increases, but increases less than treatment group prevalence, the same solution needs to apply. That is, Δ*Treatment* and Δ*Control* need to be switched.

### Limitations

I used data from Georgia for completing these analyses. With over a million student surveys from over a thousand schools, sample size was not an issue (Ruscio, 2008). One might argue that these data are not representative of the nation as a whole or for specific circumstances in which an intervention might be tested. Indeed, patterns for high school students are slightly suppressed compared to the most recent Monitoring the Future report (Johnston et al., 2018) and recent Youth Behavior Risk Surveillance Survey findings (Kann et al., 2018). Researchers with access to other datasets are encouraged to apply the tests presented in this paper to their own data to verify the conclusions I present. My analyses of *RRP* include only hypothetical data. A real-world test of *RRP* has yet to be completed.

Because *RRP* is a risk ratio, it has inherent limitations that researchers should be aware of. Effect size statistics are commonly thought of as being estimates that are independent of sample size. However, results from small samples may yield unreliable outcomes. Base rates and rates of change may also affect the performance of *RRP*. For example, very small pretest–posttest changes in treatment and control conditions may yield spurious findings. Future development may consider a means for estimating confidence intervals.

Interpreting *RRP* outcomes must always be considered in light of other considerations. *RRP* values should always be presented along with prevalence data. While a valuable alternative, I strongly advice using *RRP* alongside descriptions of prevalence rates, Skara–Sussman *Percentage Reductions*, and Cohen’s effect size statistics.

## Footnotes

1 Cohen’s *h* and Cohen’s *d* produce identical results for these data.
